# The complete chloroplast genome sequence of *Hemerocallis fulva*

**DOI:** 10.1080/23802359.2020.1829126

**Published:** 2020-10-09

**Authors:** Yan Zheng, Jingling Li, Haimei Chen, Linfang Huang

**Affiliations:** a Key Research Laboratory of Traditional Chinese Medicine Resources Protection, Administration of Traditional Chinese Medicine, National administration of Traditional Chinese Medicine, Institute of Medicinal Plant Development, Chinese Academy of Medical Sciences, Peking Union Medical College, Beijing, China; b Jiangxi University of Traditional Chinese Medicine, Nanchang, Jiangxi; c Southwest University, Chongqing, China

**Keywords:** Chloroplast genome, *Hemerocallis fulva*, phylogenetic analysis

## Abstract

*Hemerocallis fulva* L. is a traditional Chinese medicine. The flowers of *H. fulva* are used in ethnic medicine to treat various diseases, including certain central nervous system diseases. In this study, we characterized the complete chloroplast genome of *H. fulva*. It is 156,059 bp in length and encodes 87 protein-coding genes, 38 transfer RNA (tRNA) genes, and 8 ribosomal RNA (rRNA) genes. The phylogenomic analysis showed that the *H. fulva* and species of *Anemarrhena asphodeloides Bunge*, *Liriope muscari*, and *Liriope spicata* were clustered together. This chloroplast genome sequencing offers genetic background for conservation and phylogenetic studies.

*Hemerocallis fulva* L.belongs to the Liliaceae family, which is widely used in folk emotional health improvement drugs in East Asia (China, Japan) and North America (Lin et al. 2011). Its flower has antioxidation (Lin et al. 2011), antibacterial (Sarg et al. 1990), antitumor (Cichewicz et al. 2004), and sleep improvement effects (Uezu 1998). Because of *H. fulva* flower is rich of hyperin (Guo et al. 2013), it is a promising antidepressant drug (Zheng et al. 2012).

However, as a result of long-cultivation and interspecific hybrids, there is confusion in the classification of the genus *Hemerocallis* based on phenotypic characterization. For example, wild *Hemerocallis* always showed a single flower color, whereas modern hybrid horticultural varieties always showed a more complex color distribution pattern (Cui et al. 2019). To provide a scientific classification way to *Hemerocallis* L., we conducted a chloroplast genome research and a phylogenetic analysis of *H. fulva.*

Genomic DNA was extracted from fresh leaves of a seedling of *H. fulva* from Huazhong Medicinal Botanical Garden, Institute of Chinese Medicinal Materials, Hubei Academy of Agricultural Sciences (Hubei, China, N30.180978, E109.756823). Genomic DNA was extracted with plant genomic DNA kit (Tiangen Biotech, China) and sequenced by using the Hiseq 2500 platform (Illumina, San Diego, CA). The chloroplast genome was assembled from the raw sequence data by using NOVOPlasty (v.2.7.2) with the seed sequence of *rbc*L from *Arabidopsis thaliana* (Dierckxsens et al. 2017). By using Bowtie 2 (v.2.0.1) (Langmead et al. 2009) to map all the original reads to the assembly, the correctness of the assembly is verified under the default settings. The annotation of the chloroplast genome was originally performed using CPGAVAS2 (Shi et al. 2019) and then edited using Apollo (Misra and Harris 2006). The genome sequence and annotations have been deposited in GenBank with accession number MT806177.

The size of the chloroplast genome of *H. fulva* is 156,059 bp, including a large single-copy (LSC) region of 84,826 bp and a small single-copy (SSC) region of 18,495 bp separated by a pair identical inverted repeat regions (IRs) of 26,369 bp each. A total of 133 genes were successfully annotated containing 87 protein-coding genes, 38 tRNA genes, and 8 rRNA genes. And the GC content of the three regions is 43, 35, and 32% for IRs, LSC, and SSC, respectively, indicating that IR has the highest GC content. Among them, 11 protein-coding genes had one intron, and 3 protein-coding genes had two introns. 8 tRNA genes were found to contain one intron. To reveal the phylogenetic position of *H. fulva* with other members of Liliaceae Juss., a phylogenetic analysis was performed based on 21 complete chloroplast genomes from the Liliaceae family. *Smilax nipponica* (NC_049024) and *Smilax china* (NC_049022) were set as the outgroups. The MAFFT (7.037 version) (Katoh and Standley 2013) was used to extract the coding sequences, and a total of 61 coding sequences (*acc*D, *atp*A, *atp*B, *atp*E, *atp*F, *atp*H, *atp*I, *ccs*A, *cem*A, *clp*P, *mat*K, *pet*A, *pet*B, *pet*D, *pet*G, *pet*L, *pet*N, *psa*A, *psa*B, *psa*C, *psa*I, *psa*J, *psb*A, *psb*C, *psb*D, *psb*E, *psb*F, *psb*H, *psb*I, *psb*K, *psb*L, *psb*M, *psb*T, *rbc*L, *rpl*2, *rpl*14, *rpl*16, *rpl*20, *rpl*22, *rpl*23, *rpl*32, *rpl*33, *rpl*36, *rpo*A, *rpo*B, *rpo*C1, *rpo*C2, *rps*2, *rps*3 *rps*4, *rps*7, *rps*8, *rps*11, *rps*12, *rps*14, *rps*16, *rps*18, *rps*19, *ycf*2*,ycf*3, *ycf*4) were presented in all of the 23 species. Then the MAFFT (7.037 version) was used to concatenate the coding sequences and align the concatenation sequences. RAxML (version 8.2.12) (Stamatakis 2014) was used to construct the maximum likelihood (ML) tree; bootstrap probability values were calculated from 1000 replicates. The phylogenetic tree shows that the *H. fulva* and species of *Anemarrhena asphodeloides Bunge*, *Liriope muscari*, and *Liriope spicata* were clustered together. In this article, we report the complete chloroplast genome of *H. fulva*, which will provide useful genetic resources for further studying on genetic diversity of this important species and theoretical reference for the classification of *Hemerocallis* L (Figure 1).

**Figure 1.**
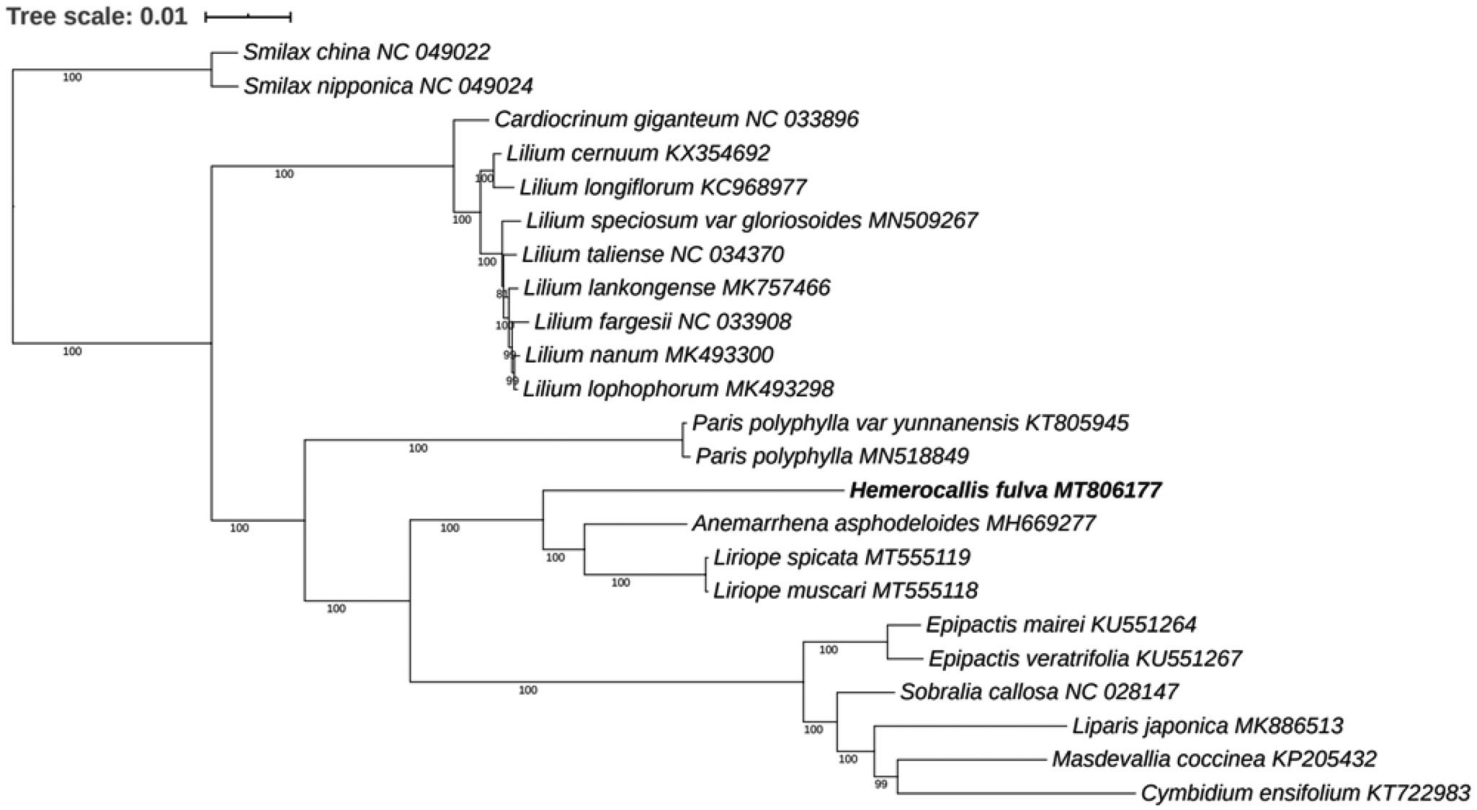
The phylogenetic tree of *H. fulva* and its closest relatives. *Smilax nipponica* (NC_049024) and *Smilax china* (NC_049022) were set as the outgroups. The number on the branch indicates the bootstrap value.

## Data Availability

The data that support the findings of this study are openly available in NCBI at https://www.ncbi.nlm.nih.gov/, the accession number is MT806177, and raw sequencing data used in this study have been deposited in SRA with accession number SRR12506380.
